# Arterial Stiffness: Its Relation with Prediabetes and Metabolic Syndrome and Possible Pathogenesis

**DOI:** 10.3390/jcm10153251

**Published:** 2021-07-23

**Authors:** Juan J. Gagliardino, Martin R. Salazar, Walter G. Espeche, Paula E. Tolosa Chapasian, Daniela Gomez Garizoain, Ricardo D. Olano, Rodolfo N. Stavile, Eduardo Balbín, Camilo Martinez, Betty C. Leiva Sisnieguez, Carlos E. Leiva Sisnieguez, Horacio A. Carbajal

**Affiliations:** 1 CENEXA, Centro de Endocrinología Experimental y Aplicada (UNLP-CONICET-CEAS CICPBA), Facultad de Ciencias Médicas UNLP, La Plata 1900, Buenos Aires, Argentina; camilomarti@hotmail.com; 2 Unidad de Enfermedades Cardiometabólicas, Hospital General San Martín, La Plata 1900, Buenos Aires, Argentina; salazarlandea@gmail.com (M.R.S.); wespeche@gmail.com (W.G.E.); tolosachapasian@gmail.com (P.E.T.C.); danielagomezgarizoain@gmail.com (D.G.G.); olanodaniel@gmail.com (R.D.O.); nicolasstavile@yahoo.com.ar (R.N.S.); edubalbin@hotmail.com (E.B.); bceci_ls@yahoo.es (B.C.L.S.); carlosenriqueleiva@yahoo.com.ar (C.E.L.S.); hcarbaj@gmail.com (H.A.C.); 3 Facultad de Ciencias Médicas, Universidad Nacional de La Plata (UNLP), La Plata 1900, Buenos Aires, Argentina

**Keywords:** pulse wave velocity, OGTT, prediabetes, TG/HDL ratio, dyslipidemia, arterial stiffness

## Abstract

Aims: To evaluate arterial stiffness indicators in people with prediabetes (PreD) and its possible pathogenesis. Materials and methods: Pulse wave velocity (PWV) was measured in 208 people with FINDRISC ≥ 13 (57 ± 8 years old, 68.7% women) and thereafter divided into those having either normal glucose tolerance (NGT) or PreD. In each subgroup we also identified those with/out insulin resistance (IR) measured by the triglyceride/HDL-c ratio (normal cut off values previously established in our population). Clinical and metabolic data were collected for all participants. PWV was compared between subgroups using independent t test. Results: Women and men had comparable clinical and metabolic characteristics with obesity (BMI ≥ 30) and antihypertensive-statin treatment, almost half with either NGT or PreD. Whereas 48% of NGT people presented IR (abnormally high TG/HDL-c ratio), 52% had PreD. PWV was significantly higher only in those with a complete picture of metabolic syndrome (MS). Conclusions: Since PWV was significantly impaired in people with a complete picture of MS, clinicians must carefully search for early diagnosis of this condition and prescribe a healthy life-style to prevent development/progression of CVD. This proactive attitude would provide a cost-effective preventive strategy to avoid CVD’s negative impact on patients’ quality of life and on health systems due to their higher care costs.

## 1. Introduction

The prevalence of type 2 diabetes (T2D) is continuously growing worldwide [1]. Argentina is no exception: the national survey of risk factors showed that in the period 2005–2008 and the adult population, it rose from 8.4 to 12.7% (a 51% increase) [2].

Its frequent association with other cardiovascular risk factors (CVRF), combined with late diagnosis and inappropriate treatment facilitates the development and progression of chronic micro- and macrovascular complications that lower patients’ quality of life and significantly increase their cost of care [3,4].

Clinical manifestation of T2D is preceded by a state identified as pre-diabetes (PreD) characterized by blood glucose level ≤ 126 mg/dL but ≥100 mg/dL [5]. PreD is a heterogeneous state depending on the degree of impaired glucose metabolism, namely: impaired fasting glucose (IFG) s ≥ 100 ≤ 126 mg/dL, impaired glucose tolerance (IGT) with 2 h post glucose load ≥ 140 ≤ 200 mg/dL, and a combination of these two alterations. This classification is important because the annual transition rate for PreD to T2D increases from 4.66 (2.47–6.85/year in people with isolated IGT, 7.54 (4.63–10.45) in those with isolated IFG; and 12.13 (4.27–20.00) in people with a combination of IFG and IGT [6].

Notwithstanding, whereas it is widely accepted that people with PreD have a higher risk of developing cardiovascular disease (CVD) [7], it is controversial whether they currently reflect CVD markers: whereas some studies showed a composite of central and peripheral arterial stiffness [8], others did not [9]. Di Pino and et al., found a significant and strong correlation between HbA1c normal vs. altered and arterial stiffness such as augmentation pressure, augmentation index, and intima-media thickness [10], while the authors found no correlation with the PWV. However, they showed that the subjects with high 1 h post load glycemia exhibit altered markers of cardiovascular disease (PWV) [11].

Unfortunately, PreD is frequently not recognized as a real disease, and therefore its diagnosis is often underestimated and inadequately treated [12]. Our group is currently conducting a diabetes primary prevention pilot study [13]. We therefore identified people with PreD and have already reported that most are overweight/obese, show dyslipidemia, and frequently high blood pressure [14].

On account of the above mentioned evidence and apparent discrepancies, the aim of the present study is to evaluate whether people with PreD currently show indicators of CVD. This demonstration could be useful for health authorities to implement proactive PreD diagnostic strategies to ensure early diagnosis and appropriate treatment.

## 2. Material and Methods

This study included people between 45 and 75 years of age recruited for the Pilot Program for Primary Prevention of Diabetes of Buenos Aires Province (PPDBA) in the Cardiometabolic Unit of the “Hospital San Martín” (La Plata, Buenos Aires, Argentina) during the year 2018. Details of the PPDBA study were previously reported [13,14]. Briefly, it is a prospective, randomized cohort study that aims to evaluate the preventive effect of adopting healthy lifestyles (healthy meal plan and regular practice of physical activity) on the transition rate from PreD to T2D.

For recruiting patients, we used an opportunistic approach: people visiting a physician’s office for reasons other than T2D filled out the FINDRISC questionnaire [15]. Those individuals with a FINDRISC score ≥13 points (cut-off value indicated by Prof Jakko Tuomilheto, a PPDBA advisor), received an oral glucose tolerance test (OGTT), following WHO recommendations [16], recording FPG and 2 h plasma glucose. Total cholesterol, high-density lipoprotein (HDL)-cholesterol, low-density lipoprotein (LDL)-cholesterol, and triglycerides (TG) were also measured. All blood samples were processed in a single laboratory (CentraLab, CABA, Argentina) within 24 h of extraction.

These people were invited to participate in the present study and, after accepting and signing a written informed consent, we made a cardiovascular evaluation with pulse wave velocity (PWV) measurement.

An epidemiological-clinical chart including self-reported history of CVD, smoking, dyslipidemia, diabetes, use of statins and antihypertensive drugs was completed. Body weight was determined with subjects wearing light clothing and no shoes. Height was also measured without shoes, using a metallic metric tape. Body mass index (BMI) was calculated using the formula weight (Kg)/height (m) ^2^. Waist circumference was measured with a relaxed abdomen using a metallic metric tape on a horizontal plane above the iliac crest.

Three BP measurements used a validated oscillometric automatic BP device (OMRON HEM 705 CP) with cuff and bladder dimensions depending on arm circumference [17]. Measurements were made with the subject sitting, the back supported, without crossing the legs, both feet on the floor, the arm uncovered, supported, at heart level, and without speaking. Office BP was defined as an average of these three determinations; office hypertension was defined as BP ≥ 140/90 mmHg [17].

A single and highly qualified specialist, blinded to the patients’ cardiometabolic data, measured the PWV in all patients with a rest of at least 5 min in a sitting position and later in supine position resting at least 2 more minutes until all the electrodes are placed prior to the measurements. It was used for non-invasive hemodynamic measurements, previously validated device, Exxer IE device [18,19]. Carotid-femoral PWV was calculated by dividing distance traveled by transit time, with the patient resting in dorsal decubitus. Tonometers were placed on the right carotid, right radial, and right femoral arteries. Transit time was estimated by measuring from the foot of the carotid wave to the foot of the femoral wave. The device measures transit time as the time delay between the arrival of the pulse wave at the common carotid artery and the common femoral artery. The carotid and femoral pulse waves (3 serial measurements separated by approximately 1 min) were recorded simultaneously for at least one respiratory cycle (5–6 s). The carotid–femoral pathway was measured by the direct path length. Since the use of direct distance leads to overestimation of real PWV, we used a scaling factor of 0.8 derived from Sugawara et al. [20] and Weber et al. [21] to convert PWV recorded for direct distances to “real” PWV.

According to the results of the OGTT the sample population was divided dichotomically into normal glucose metabolism (FPG < 100 mg/dL and 2 h post glucose load < 140 m/dL) or having PreD, using the criteria of the American Diabetes Association [5]. As mentioned, this diagnosis included IFG, IGT, and the combination of the two metabolic dysfunctions. People who met criteria of diabetes were excluded.

Previously published cut-off points of plasma TG/HDL-C ratios of 2.5 and 3.5 (expressed both in mg/dL) [22], for women and men, respectively, were used to identify participants with insulin resistance (IR).

In each strata normal glucose metabolism and PreD, characteristics of individuals were compared between individuals with or without IR. Continuous variables were expressed as mean ± standard deviation (SD) and compared between risk groups using *t*-test for independent samples; proportions were represented as percentages and compared by χ^2^ test.

We compared PWVs of subjects with IR (abnormally high-TG/HDL ratio) to those with normal insulin sensitivity (TG/HDL ratio within normal range): people with normal glucose metabolism vs. those with PreD using independent samples t test.

Statistical analyses were performed using SPSS (SPSS, Chicago, IL). All significant tests were 2-tailed, and *p* < 0.05 were considered statistically significant.

## 3. Results

The general characteristics of our patient population separated by gender are shown in Table 1. It can be seen that they had comparable clinical and metabolic characteristics, except the expected higher serum HDL-c values in women. Overall, they have a low percentage of CVD history, a high percentage of treatment with antihypertensive drugs and some with statins, BMI compatible with obesity, a little over 50% with PreD, and under 50% with IR measured by the TG/HDL-c ratio. Accordingly, we classified the whole population sample into people with normal glucose tolerance (NGT) and those with PreD, and within each group a subgroup having or not insulin resistance (IR) (depending on whether their TG/HDL-c ratio was normal or abnormally high) (Table 2).

Although all participants had a FINDRISC score of moderate risk, 48% had NGT whereas the remaining 52% had PreD; also, within each subgroup we identified those with IR (abnormally high TG/HDL-c ratio).

In the subgroup of people with NGT, 34% had IR and no significant differences between people with/out IR were found in percentages of smokers, statin or hypertensive treatment, CVD background, SBP/DBP, or PWV values. However, the latter had significantly lower age (4 years) and HDL-c, but significantly higher serum triglycerides.

In the subgroup of people with PreD, 53% presented IR and exhibited significantly lower serum HDL-c but significantly higher serum triglyceride; they also showed significantly higher PWV a representative and significant indicator of arterial stiffness.

## 4. Discussion

Our study shows that although all subjects in our cohort had a FINDRISC score ≥ 13, i.e., moderate risk of developing T2D and cardio-metabolic disease (CVD), 48% currently had NGT, whereas the rest presented PreD. Dyslipidemia (mean high serum triglyceride and low HDL-c), were commonly present in both the NGT and the PreD subgroups. Moreover, most were obese, almost half received hypertension treatment, and some also received statins.

In contrast, while within the NGT subgroup 34% had IR whereas this condition was significantly higher (53%) in the PreD subgroup.

PWV is a well-accepted indicator of arterial stiffness, however, it is significantly present only in the latter subgroup, when PreD was associated with other clinical and metabolic abnormalities. Meanwhile, Li and et al., showed a non-significant increase in arterial stiffness, as shown by PWV, in isolated IFG subjects [23,24]. This fact suggests that the combination of high BMI, dyslipidemia, PreD, and IR was necessary to develop values higher of PWV.

CVD is considered the leading cause of mortality globally representing a major challenge to the healthcare systems [25]. Among the common cardiovascular risk factors, T2D and its preceding state, PreD, increase the risk of CVD two- to four-fold [1,12]. For that reason, major organizations such as the AACE have called attention to the importance of the diagnosis and appropriate treatment of both clinical conditions to prevent development and progression of these complications. Further, the recent EUROASPIRE V study recommends the compelling need to improve both screening for and management of patients with dysglycemia and CAD. It also mentioned that screening for both T2D and PreD is poorly implemented, though approximately two-thirds of coronary patients have these pathologies [26]. All this evidence lends support to our research and findings.

Obesity, a condition found in our population sample whose prevalence is increasing worldwide, is attaining the characteristics of an epidemic [2]. The problem is complex since excess adiposity increases the risk of CVD, T2D, and hypertension [27,28]. These clinical conditions share a state of resistance to insulin-mediated glucose disposal [29,30] that, together with compensatory hyperinsulinemia, have been shown to be independent predictors for all of them [31,32]. Further, it has been shown that in obese people weight loss is followed by a simultaneous and significant improvement in CVD risk factors and IR. Therefore, weight loss prescription will provide an effective tool to simultaneously decrease overweight/obesity, T2D and CVD frequency [33]. At present, lifestyle modification is mainstay management for people with PreD [34]. Supporting this concept, although aortic stiffness is associated with IGT and T2D in an elderly cohort in Finland, this association is reported to reduce significantly when people practice intense physical activity [35], thereby proving the efficacy of this clinical intervention [36]. Despite this evidence, identification and treatment of at-risk people with PreD is far from satisfactory [37]. Our data strengthen the idea that individuals with PreD and complete metabolic syndrome (high TG/HDL-c ratio), can reasonably receive an early pharmacologic prescription to delay the established cardiovascular damage. Thus, low salt intake and increased vegetables in the diet, smoking cessation, and increasing physical activity are recommendations that these individuals should have early [37].

On account of this situation and to facilitate identification of people with higher CVD risk, the World Health Organization, the Adult Treatment Panel III, and the International Diabetes Federation created a new diagnostic category, the metabolic syndrome having a specific abnormality: insulin resistance. A long-standing hypothesis is that insulin resistance correlates positively with development of some degree of glucose intolerance, high triacylglycerol and low HDL, essential hypertension, procoagulant and proinflammatory states, altogether increasing the risk of CVD [38].

CVD is the leading cause of death worldwide [39], arterial stiffness being an independent risk factor contributing to its development and progression [40].

Arterial stiffness is associated with components of the cardiometabolic syndrome, a combination of interactive cardiac and metabolic risk factors including overweight/obesity, hypertension, insulin resistance/hyperinsulinemia, dyslipidemia, and other metabolic impairments [41,42]. All these conditions were clearly present in our subpopulation of PreD plus IR together with significantly higher PWV, thereby supporting their real existence and interrelation.

Since carotid-femoral pulse wave velocity measurement is accepted as a valid non-invasive method to determine arterial stiffness (Class I, Level of Evidence A) [43], the American Heart Association has recommended its measurement to improve and standardize vascular research on arterial stiffness [44]. Complementarily, they also suggested that when a standardized insulin assay is not available, a useful approach to identify people with IR is to use the cut-off points for the TG/HDL-c ratio [45,46]. For this reason, we previously established the cut-off points for this ratio in normal people in our location [22].

**Figure 1:**
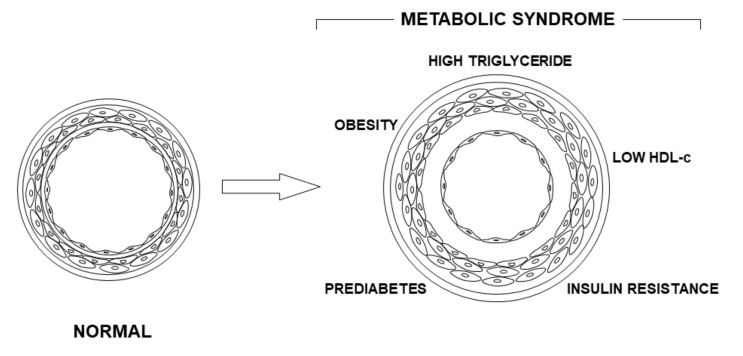
Postulated pathogenesis of the arterial stiffness. The figure shows the normal wall artery structure (**left**) and another with abnormal stiffness (**right**). The later shows an increased vascular diameter together with hypertrophy, a proliferation of smooth muscle cells, and an increased fibrosis of the intima layer.

These tools enabled us to demonstrate that people with PreD associated with clinical and metabolic indicators of metabolic syndrome showed clear indicators of increased arterial stiffness, namely, significantly delayed PWV. This postulated pathogenic mechanism is represented in Figure 1.

Although values higher PWV time was clearly found in our people with PreD and IR, this data might be considered with caution due to: (a) The relatively small number of cases; (b) the people in the subgroup with NGT and IR were significantly younger than their counterparts without IR; and (c) many patients were in treatment with cardiovascular drugs that could potentially modify the results.

Regarding this last point however, we found no percentage differences between patients using statin versus antihypertensive drugs in subgroups of people with/out IR, and PWV differences also remained significant after adjustment for those treatments.

In brief, we have demonstrated that PWV was significantly impaired in people with the whole picture of metabolic syndrome. Therefore, clinicians must carefully search for early diagnosis and prescription of healthy life-style to prevent the development/progression of CVD. This proactive attitude might provide a cost-effective preventive strategy to avoid CVD’s negative impact on patients´ quality of life and on health systems due to higher care costs.

## Figures and Tables

**Table 1:** Clinical, metabolic, and therapeutic characteristics of the cohort according to gender.

	Women *n* = 141	Men *n* = 67	*p* *
Age, years (mean ± SD)	57 ± 7	58 ± 8	0.137
CVD antecedents (%)	6.4	10.4	0.304
Current smoking (%)	9.2	14.9	0.220
Antihypertensive drugs use (%)	46.8	38.8	0.278
Statins drugs use (%)	11.3	14.9	0.466
BMI, kg/m^2^ (mean ± SD)	30.3 ± 5.2	29.8 ± 5.5	0.585
Office systolic, BP mmHg (mean ± SD)	134 ± 16	139 ± 15	0.083
Office diastolic, BP mmHg (mean ± SD)	82 ± 11	84 ± 10	0.176
Fasting glucose, mg/dL (mean ± SD)	99 ± 13	101 ± 12	0.280
Prediabetes (%)	51.1	53.7	0.129
LDL-cholesterol, mg/dL (mean ± SD)	125 ± 38	119 ± 33	0.329
Triglycerides, mg/dL (mean ± SD)	143 ± 77	143 ± 69	0.982
HDL-cholesterol, mg/dL (mean ± SD)	53 ± 12	45 ± 11	<0.001
High TG/HDL ratio (%) (IR indicator)	46.1	38.8	0.322

* Continuous variables were compared using *t*-test for independent samples and proportions using χ^2^ test. HDL: high-density lipoprotein; LDL: low-density lipoprotein; BP: Blood pressure; BMI: body mass index; CVD: cardiovascular disease; TG/HDL: triglyceride/high-density lipoprotein cholesterol.

**Table 2:** Population according to OGTT and Insulin resistance (measured by TG/HDL-c Ratio).

	Normal Glucose Metabolism			PreDM		
	W/Out IR *n* = 66	With IR *n* = 34 34%	*p* *	W/Out IR *n* = 51	With IR *n* = 57 53%	*p* *
Age (years), mean ± SD	58 ± 7	54 ± 7	0.008	57 ± 7	58 ± 8	0.222
Women, (%)	65.2	76.5	0.246	64.7	68.4	0.683
Current smokers, (%)	12.1	11.8	0.959	13.7	7.0	0.250
Treatment with statin, (%)	12.1	5.9	0.325	13.7	15.8	0.763
Treatment with antihypertensives (%)	37.9	52.9	0.150	41.2	49.1	0.408
Antecedent of CVD (%)	13.6	8.8	0.483	2.0	5.3	0.364
BMI (kg/m^2^), mean ± SD	28.1 ± 4.4	30.5 ± 5.4	0.027	30.1 ± 5.4	32.1 ± 5.4	0.062
Systolic BP (mmHg), mean ± SD	134 ± 19	135 ± 15	0.754	135 ± 14	139 ± 15	0.203
Diastolic BP (mmHg), mean ± SD	82 ± 13	82 ± 10	0.964	82 ± 10	84 ± 11	0.503
FPG (mg/dL), mean ± SD	89 ± 9	92 ± 10	0.140	107 ± 9	108 ± 9	0.640
LDL-Cholesterol (mgl/L), mean ± SD	122 ± 33	122 ± 33	0.990	118 ± 40	128 ± 41	0.182
Triglycerides (mgl/L), mean ± SD	100 ± 27	185 ± 70	<0.001	102 ± 33	204 ± 85	<0.001
HDL-Cholesterol (mgl/L), mean ± SD	55 ± 9	42 ± 9	0.001	57 ± 14	43 ± 9	<0.001
Pulse wave velocity (m/sec), mean ± SD	7.7 ± 2.0	7.9 ± 2.0	0.615	7.4 ± 1.8	8.3 ± 2.3	0.024

* Continuous variables were compared using *t*-test for independent samples and proportions using χ^2^ test. PreD: prediabetes; IR: insulin resistance; HDL: high-density lipoprotein; LDL: low-density lipoprotein; BP: Blood pressure; FPG: Fasting glucose; BMI: body mass index; CVD: cardiovascular disease; TG/HDL: triglyceride/high-density lipoprotein cholesterol ratio.

## Data Availability

The data presented in this study are available on request from the corresponding author.

## References

[1] International Diabetes Federation (2019). IDF Diabetes Atlas.

[2] Instituto Nacional de Estadísticas y Censos (INDEC) Resultados de la 4ta Encuestan Nacional de Factores de Riesgo. http://www.msal.gob.ar/images/stories/bes/graficos/0000001622cnt-2019-10_4ta-encuesta-nacional-factores-riesgo.pdf.

[3] Elgart J.F., Asteazarán S., De La Fuente J.L., Camillucci C., Brown J.B., Gagliardino J.J. (2014). Direct and indirect costs associated to type 2 diabetes and its complications measured in a social security institution of Argentina. Int. J. Public Health.

[4] Gagliardino J., Aschner P., Baik S., Chan J., Chantelot J., Ilkova H., Ramachandran A. (2012). Patients’ education, and its impact on care outcomes, resource consumption and working conditions: Data from the International Diabetes Management Practices Study (IDMPS). Diabetes Metab..

[5] American Diabetes Association (2017). Classification and Diagnosis of Diabetes: Standards of Medical Care in Diabetes-2018. Diabetes Care.

[6] Gerstein H.C., Santaguida P., Raina P., Morrison K., Balion C., Hunt D., Yazdi H., Booker L. (2007). Annual incidence and relative risk of diabetes in people with various categories of dysglycemia: A systematic overview and meta-analysis of prospective studies. Diabetes Res. Clin. Pr..

[7] Færch K., Vistisen D., Johansen N.B., Jørgensen M.E. (2014). Cardiovascular Risk Stratification and Management in Pre-Diabetes. Curr. Diabetes Rep..

[8] Loehr L., Meyer M., Poon A.K., Selvin E., Palta P., Tanaka H., Pankow J., Wright J.D., Griswold M.E., Wagenknecht L.E. (2016). Prediabetes and Diabetes Are Associated with Arterial Stiffness in Older Adults: The ARIC Study. Am. J. Hypertens..

[9] Webb D.R., Khunti K., Silverman R., Gray L.J., Srinivasan B., Lacy P.S., Williams B., Davies M. (2010). Impact of metabolic indices on central artery stiffness: Independent association of insulin resistance and glucose with aortic pulse wave velocity. Diabetologoa.

[10] Di Pino A., Scicali R., Calanna S., Urbano F., Mantegna C., Rabuazzo A.M., Purrello F., Piro S. (2014). Cardiovascular Risk Profile in Subjects with Prediabetes and New-Onset Type 2 Diabetes Identified by HbA1cAccording to American Diabetes Association Criteria. Diabetes Care.

[11] Di Pino A., Urbano F., Scicali R., Di Mauro S., Filippello A., Scamporrino A., Piro S., Purrello F., Rabuazzo A.M., Pino (2019). 1 h Postload Glycemia Is Associated with Low Endogenous Secretory Receptor for Advanced Glycation End Product Levels and Early Markers of Cardiovascular Disease. Cells.

[12] Zand A., Ibrahim K., Patham B. (2018). Prediabetes: Why Should We Care?. Methodist Debakey Cardiovasc. J..

[13] Gagliardino J.J., Etchegoyen G., Bourgeois M., Fantuzzi G., García S., González L., Elgart J.F., Ré M., Ricart A., Ricart J.P. (2016). Prevención primaria de diabetes tipo 2 en Argentina: Estudio piloto en la provincia de Buenos Aires. Rev. Argent Endocrinol. Metab..

[14] Gagliardino J.J., Elgart J.F., Bourgeois M., Etchegoyen G., Fantuzzi G., Ré M., Ricart J.P., García S., Giampieri C., González L. (2018). Diabetes primary prevention program: New insights from data analysis of recruitment period. Diabetes Metab. Res. Rev..

[15] Lindström J., Tuomilehto J. (2003). The Diabetes Risk Score: A practical tool to predict type 2 diabetes risk. Diabetes Care.

[16] WHO Consultation (1999). Definition, Diagnosis and Classification of Diabetes Mellitus and Its Complications. Part 1: Diagnosis and Classification of Diabetes Mellitus.

[17] Williams B., Mancia G., Spiering W., Agabiti Rosei E., Azizi M., Burnier M., Clement D.L., Coca A., De Simone G., Dominiczak A. (2018). 2018 ESC/ESH Guidelines for the management of arterial hypertension. Eur. Heart J..

[18] Cuffaro P.E., Morales M.S., Barochiner J., Rada M.A., Alfie J., Aparicio L.S., Galarza C.R., Micali R.G., Marin M.J., Waisman G.D. (2018). Validation of a new piezoelectric device for noninvasive measurement of central aortic systolic blood pressure. Blood Press. Monit..

[19] Olano R.D., Espeche W.G., Salazar M.R., Forcada P., A Chirinos J., De Iraola A., Sisnieguez C.E.L., Sisnieguez B.C.L., Balbín E., Carbajal H.A. (2019). Evaluation of ventricular-arterial coupling by impedance cardiography in healthy volunteers. Physiol. Meas..

[20] Sugawara J., Hayashi K., Yokoi T., Tanaka H. (2008). Age-Associated Elongation of the Ascending Aorta in Adults. JACC Cardiovasc. Imaging.

[21] Weber T., Ammer M., Rammer M., Adji A., O’Rourke M.F., Wassertheurer S., Rosenkranz S., Eber B. (2009). Noninvasive determination of carotid–femoral pulse wave velocity depends critically on assessment of travel distance: A comparison with invasive measurement. J. Hypertens..

[22] Salazar M.R., Carbajal H.A., Espeche W.G., Sisnieguez C.E.L., Balbín E., Dulbecco C.A., Aizpurúa M., Marillet A.G., Reaven G.M. (2012). Relation among the Plasma Triglyceride/High-Density Lipoprotein Cholesterol Concentration Ratio, Insulin Resistance, and Associated Cardio-Metabolic Risk Factors in Men and Women. Am. J. Cardiol..

[23] Li C.-H., Wu J.-S., Yang Y.-C., Shih C.-C., Lu F.-H., Chang C.-J. (2012). Increased Arterial Stiffness in Subjects with Impaired Glucose Tolerance and Newly Diagnosed Diabetes but Not Isolated Impaired Fasting Glucose. J. Clin. Endocrinol. Metab..

[24] Li C.-H., Lu F.-H., Yang Y.-C., Wu J.-S., Chang C.-J. (2019). Increased Arterial Stiffness in Prediabetic Subjects Recognized by Hemoglobin A1c with Postprandial Glucose but Not Fasting Glucose Levels. J. Clin. Med..

[25] World Health Organization The Top 10 Causes of Death [Internet]. 24 May 2018. https://www.who.int/news-room/fact-sheets/detail/the-top-10-causes-of-death.

[26] Ferrannini G., De Bacquer D., De Backer G., Kotseva K., Mellbin L., Wood D., Rydén L. (2020). Screening for Glucose Perturbations and Risk Factor Management in Dysglycemic Patients with Coronary Artery Disease—A Persistent Challenge in Need of Substantial Improvement: A Report From ESC EORP EUROASPIRE V. Diabetes Care.

[27] West K.M., Kalbfleisch J.M. (1971). Influence of Nutritional Factors on Prevalence of Diabetes. Diabetes.

[28] Wilson P.W., D’Agostino R.B., Sullivan L., Parise H., Kannel W.B. (2002). Overweight and obesity as determinants of cardiovascular risk: The Framingham experience. Arch. Intern. Med..

[29] Reaven G.M. (1988). Role of insulin resistance in human disease. Diabetes.

[30] Reaven G.M. (2003). The insulin resistance syndrome. Curr. Atheroscler. Rep..

[31] Facchini F.S., Hua N., Abbasi F., Reaven G.M. (2001). Insulin resistance as a predictor of age related diseases. J. Clin. Endocrinol. Metab..

[32] Zavaroni I., Bonini L., Gasparini P., Barilli A.L., Zuccarelli A., Dall’Aglio E., Delsignore R., Reaven G.M. (1999). Hyperinsulinemia in a normal population as a predictor of non-insulin-dependent diabetes mellitus, hypertension, and coronary heart disease: The Barilla factory revisited. Metabolism.

[33] Reaven G.M. (2008). Insulin Resistance: The Link between Obesity and Cardiovascular Disease. Endocrinol. Metab. Clin. N. Am..

[34] Huang Y., Cai X., Mai W., Li M., Hu Y. (2016). Association between prediabetes and risk of cardiovascular disease and all cause mortality: Systematic review and meta-analysis. BMJ.

[35] Stratmann B., Rydzkowski M., Tschoepe D. (2017). Pulse Wave Velocity Rather than Atherosclerotic Markers Describe Vascular Status of Patients with Type 2 Diabetes Mellitus. Diabetes.

[36] Metsämarttila E., Rodilla E., Jokelainen J., Herrala S., Leppäluoto J., Keinänen-Kiukaanniemi S., Herzig K.-H. (2018). Effect of physical activity on pulse wave velocity in elderly subjects with normal glucose, prediabetes or Type 2 Diabetes. Sci. Rep..

[37] Brannick B., Dagogo-Jac S. (2018). Prediabetes and Cardiovascular Disease Pathophysiology and Interventions for Prevention and Risk Reduction. Endocrinol. Metab. Clin. N. Am..

[38] Reaven G.M. (2006). The metabolic syndrome: Is this diagnosis necessary?. Am. J. Clin. Nutr..

[39] Dugani S., Gaziano T.A. (2016). 25 by 25: Achieving Global Reduction in Cardiovascular Mortality. Curr. Cardiol. Rep..

[40] Aroor A.R., DeMarco V., Jia G., Sun Z., Nistala R., Meininger G.A., Sowers J. (2013). The role of tissue 540 Renin-Angiotensin-aldosterone system in the development of endothelial dysfunction and arterial stiffness. Front. Endocrinol..

[41] Aroor A.R., Mandavia C.H., Sowers J.R. (2012). Insulin Resistance and Heart Failure: Molecular Mechanisms. Heart Fail. Clin..

[42] Aroor A.R., Jia G., Sowers J.R. (2018). Cellular mechanisms underlying obesity-induced arterial stiffness. Am. J. Physiol. Integr. Comp. Physiol..

[43] O’Rourke M.F., Staessen J.A., Vlachopoulos C., Duprez D., Plante G.E. (2002). Clinical applications of arterial stiffness; definitions and reference values. Am. J. Hypertens..

[44] Townsend R.R., Wilkinson I.B., Schiffrin E.L., Avolio A.P., Chirinos J.A., Cockcroft J.R., Heffernan K.S., Lakatta E.G., McEniery C.M., Mitchell G.F. (2015). Recommendations for Improving and Standardizing Vascular Research on Arterial Stiffness: A Scientific Statement from the American Heart Association. Hypertension.

[45] McLaughlin T., Abbasi F., Cheal K., Chu J., Lamendola C., Reaven G. (2003). Use of metabolic markers to identify overweight individuals who are insulin resistant. Ann. Intern. Med..

[46] McLaughlin T., Reaven G., Abbasi F., Lamendola C., Saad M., Waters D., Simon J., Krauss R.M. (2005). Is there a simple way to identify insulin-resistant individuals at increased risk of cardiovascular disease?. Am. J. Cardiol..

